# Epidemiological, clinical and genotypic features of human Metapneumovirus in patients with influenza-like illness in Senegal, 2012 to 2016

**DOI:** 10.1186/s12879-019-4096-y

**Published:** 2019-05-22

**Authors:** Mamadou Malado Jallow, Amary Fall, Davy Kiori, Sara Sy, Déborah Goudiaby, Mamadou Aliou Barry, Malick Fall, Mbayame Ndiaye Niang, Ndongo Dia

**Affiliations:** 1 Département de Virologie, Institut Pasteur de Dakar, 36, Avenue Pasteur, B.P. 220, Dakar, Sénégal; 2 Institut Pasteur de Dakar, Unité d’Epidémiologie des maladies infectieuses, Dakar, Sénégal; 3 Département de Biologie, Animale Faculté des Sciences et Techniques Université Cheikh Anta DIOP de Dakar, Dakar, Sénégal

**Keywords:** Human metapneumovirus, Influenza-like illness, Epidemiology

## Abstract

**Background:**

Human metapneumovirus (HMPV) is a causal agent of acute respiratory infection, especially in primarily children. At the clinical level, HMPV is associated to several diseases including bronchitis, croup, pneumonia, bronchiolitis, reactive airway disease, chronic obstructive pulmonary disease and asthma exacerbations, specifically in children less than 5 years. Here, we carried out a retrospective pilot study, based on the processing of nasopharyngeal swabs, with a focus on the epidemiology and molecular characteristics of HMPV in Senegal.

**Methods:**

This retrospective study was conducted from January 2012 to December 2016. Briefly, all outpatients presenting to healthcare sentinel sites were screened for surveillance enrollment and included if they met criteria for ILI. Naso-oropharyngeal swabs were collected from eligible participants. For viral respiratory pathogens detection, including HMPV, the Anyplex™ II RV16 Detection kit was used. A fragment of the hMPV F gene was targeted for sequencing.

**Results:**

In total, 8209 patients with ILI were enrolled. Half of them (49.7%) were children under 5 years. Fever was the most common symptom followed by cough, and rhinitis. Three hundred eight patients were positive for HMPV (3.75%). 89 (28.9%) were detected as single infection. In co-infection cases, the most common co-infecting viruses were influenza, adenovirus and rhinovirus. HMPV detection rates in the different age groups varied significantly with the children under 5 years group accounting for 71.7% of positive patients. The temporal distribution pattern for HMPV infection showed a clear seasonal pattern with a higher activity during the rainy period (July–September). Phylogenetic analyses revealed that HMPV specimens circulating in Senegal were distributed into the two main genetic lineages, A and B. We also noted a co-circulation of both genetic lineages during the whole study period except in 2014.

**Conclusion:**

In summary, the present study characterized the recent prevalence, seasonality and genetic diversity of HMPV in a large outpatient population presented with ILI in Senegal between 2012 and 2016. Globally our results show a clear seasonal circulation pattern of HMPV in Senegal. Our findings identified children less than 5 years as more susceptible group to HMPV infection. Molecular studies identified A2, B1 and B2 as the major genotypes circulating.

## Background

Human metapneumovirus (HMPV) was first observed in isolates of nasopharyngeal aspirate samples collected from children with lower respiratory tract infection during the 1990s in Netherlands [1]. It is an enveloped, non-segmented, negative-sense, single-stranded RNA virus, and the first member of the new *Metapneumovirus* genus belonging to the *Paramyxoviridae* family that infects humans [2]. HMPV is a causal agent of acute respiratory infection (ARI) in primarily children [3]. The virus causes substantial morbidity especially in the extremes of age and in the immunocompromised [4]. For instance, in hospitalized patients younger than 5 years of age, 5-7% of acute respiratory infections worldwide can be attributed to hMPV [5]. At the clinical level, hMPV is associated to several diseases including bronchitis, croup, pneumonia, bronchiolitis, reactive airway disease, chronic obstructive pulmonary disease and asthma exacerbations, specifically in children less than 5 years of age [6]. Despite the growing burden of diseases imputed to this pathogen, there is no specific prophylactic vaccination strategy in place although various vaccine candidates are currently under investigation [7]. In addition, little is known about the epidemiology of this virus, especially in low income countries due to the low availability of molecular diagnostic tools.

To address this, we carried out a retrospective pilot study, based on the processing of nasopharyngeal swabs, with a focus on the epidemiology and molecular characteristics of HMPV in Senegal after 5 consecutive years of surveillance. We also evaluated the genetic variability in the Fusion protein (F) gene of HMPV detected. Phylogenetic analysis based on the F gene was performed to establish the relationships between Senegalese strains and previously described genotypes.

## Methods

### Study population and patient data collection

This retrospective study was conducted from January 2012 to December 2016. It should be noted that samples were collected in a sentinel flu monitoring context. Senegal, a country with around 16 Million people and situated in the western part of Africa between the Atlantic Ocean and the Sahel, had a National Influenza Center since 1974. The NIC is hosted at the Institut Pasteur Dakar (IPD) and had been part of the World Health Organization (WHO) Global Influenza Surveillance Network since 1996 [8]. The purpose of this influenza surveillance has traditionally been the early detection of influenza epidemics in the community, the identification of predominant circulating influenza strain, and the issuing of public health recommendations. In 2012, the network has been consequently enhanced with epidemiological data collection, additional sentinel sites, and virological surveillance extended to other respiratory viruses diagnosis [9].

Briefly, all outpatients presenting to healthcare sentinel sites were screened for Influenza-like Illness (ILI) surveillance. ILI was defined according to the Centers for Disease Control and Prevention (CDC) case definition: sudden onset of fever (≥38 °C) with cough or sore throat fewer than 3 days in duration. Naso-oropharyngeal swabs were collected from eligible participants, placed in universal viral transport medium (Becton Dickinson and company, Italy), stored at 4-8°C, and then transported to the NIC within the following 72 h for the testing. In addition, demographic and clinical data were collected from each patient. Once in the laboratory, specimens were processed immediately for virus detection, identification, and characterization. Aliquots of each sample were also stored at − 80 °C for biobanking or additional analysis.

### Detection of respiratory viruses

Total nucleic acid (DNA/RNA) extraction was performed from 200 μl of each sample using the QIAamp Viral RNA kit (QIAGEN, Valencia, CA, USA) according to the manufacturer’s instructions. For viral respiratory pathogens detection, the Anyplex™ II RV16 Detection kit (Seegene, Seoul, Korea) was used. The multiplex kit system enabled simultaneous detection of several respiratory viruses: influenza A virus, influenza B virus, human respiratory syncytial virus (A and B), human adenovirus, human metapneumovirus, human coronaviruses (229E, NL63 and OC43), human parainfluenza viruses (1, 2, 3 and 4), human rhinovirus (A, B and C), human enteroviruses and human bocavirus.

### Human Metapneumovirus molecular characterization and phylogenetic analysis

For the sequencing, 100 samples (20 per year) were basically selected upon three criteria: positivity for HMPV in real-time Polymerase Chain Reaction (PCR), year and week distribution of HMPV positive samples, and Ct-values values detection (lowest Ct-values are correlated with important viral load).

Viral RNA from HMPV-positive samples was extracted and reverse transcribed to cDNA using RevertAid First Strand cDNA Synthesis Kit (Thermo Scientific, Lithuania). A fragment of the hMPV F gene was targeted and amplified by nested PCR. The first amplification targeting a 750 bp fragment long was carried out using primers previously described [10]. The Phusion High-Fidelity PCR Master Mix (New England Biolabs, Ipswich MA, USA) was used for amplifications. For each sample, PCR was carried out in a total reaction volume of 50 μl consisting of 15 μl H2O RNase free, 2.5 μl of each primer (diluted at 10 μM), 25 μl of 2X Phusion Master Mix and 5 μl of cDNA template under the following cycling conditions: initial denaturation step of 15 min at 95 °C, followed by 35 PCR cycles including 30 s at 95 °C, 60 s at 55 °C, 60 s at 72 °C, and a final elongation step at 72 °C for 10 min. For the nested PCR, which targets 610 bp, internal primers hMPVF2F (5 ‘ACATGCCAACATCTGCAGGACAAATAAAAC-3’) and hMPVF2R (5′-ACATGCTGT TCACCTTCAACTTTGC-3 ‘), and 1 μl of the first PCR product were used.

For each PCR product, 5 μl mixed with 1 μl of 10X 5PRIME loading dye were loaded onto a 1% agarose gel and co-electrophoresed with appropriated molecular weight markers (100 bp ladder, New England Biolabs), before ethidium bromide (0.5 μg/ml) staining and visualization under UV.

For positive samples (610 bp size band), amplicons were cut and purified using the Gene-JET Gel Extraction Kit (Thermo Scientific), and then sent for sequencing to Beckman Coulter Services. Sequencing was performed in both directions with primers used in the Nested-PCR (hMPVF2F and hMPVF2R). Data in FASTA format were then sent to the laboratory for analysis.

Sequences successfully obtained were aligned with sequences of HMPV F gene retrieved from the GenBank database using the BioEdit Sequence alignment Editor tool [11] with the ClustalW Multiple Alignment software. Phylogenetic trees were constructed by the neighbor-joining method based on the Kimura two-parameter model in MEGA 7 [12]. The reliability and robustness of the branching orders were analyzed by bootstrap analysis of 1000 replicates. Only bootstrap replicates with values ≥70 are shown on the tree.

### Statistical analysis

The demographic and clinical characteristics of the study population and the positive cases have been analyzed by using chi-square test or Fisher’s exact test. *P* value *<* 0.05 was considered statistically significant and the 0 ± 5 year age group was used as reference group. The R.3.0.1 tool was used to perform the analyses.

### Ethical considerations

The Senegalese National Ethical Committee of the Ministry of Health approved the surveillance protocol as less than minimal risk research, and written consent forms were not required. Throughout the study, the database was shared with the Epidemiology Department at the Senegalese Ministry of Health and Prevention for appropriate public health action.

## Results

### Patient characteristics

In total, 8209 patients with ILI, ranging from 1 month to 95 years old were enrolled during the study period (Table 1): 1213 were collected in 2012 (14.8%), 1519 in 2013 (18.5%), 1931 in 2014 (23.5%), 1718 in 2015 (20.9%) and 1828 in 2016 (22.3%). The male to female ratio was 0.99 (4065/4107) with a mean age of 11 years 4 months, and a median age of 4 years 4 months. Regarding the age, nearly half of the overall patients (49.7%) were children under 5 years of age followed by 5-10 years age group with 13% (1071/8209) and 25-50 years age group with 10.7% (878/8209). Patients above 50 years old represented only 3.5% (287/8209) of enrolled patients, and for 537 (6.5%) patients the age was not documented.

**Table 1:** Clinical and Demographic Characteristics of Patients with Influenza like Illness in Senegal between 2012 and 2016

Years Total tested	2012	2013	2014	2015	2016	Total
	*N = 1213*	*N = 1519*	*N = 1931*	*N = 1718*	*N = 1828*	*N = 8209*
Characteristics						
Gender, n(%)						
Female	587 (48.39)	767 (50.49)	987 (51.11)	844 (49.13)	922 (50.44)	4107 (50.03)
Male	610 (50.29)	744 (48.98)	937 (48.52)	873 (50.81)	901 (49.29)	4065 (49.52)
Missing	16 (1.32)	8 (0.53)	7 (0.36)	1 (0.06)	5 (0.27)	37 (0.45)
Age group, n(%)						
0–5 yrs	705 (58.12)	708 (46.61)	880 (45.57)	771 (44.88)	1019 (55.74)	4083 (49.74)
5–10 yrs	140 (11.54)	179 (11.78)	218 (11.29)	287 (16.70)	246 (13.46)	1071 (13.05)
10-15 yrs	77 (6.35)	106 (6.98)	150 (7.77)	124 (7.22)	149 (8.15)	606 (7.38)
15-25 yrs	78 (6.43)	125 (8.23)	227 (11.75)	164 (9.54)	153 (8.37)	747 (9.1)
25-50 yrs	62 (5.11)	124 (8.16)	282 (14.60)	227 (13.21)	183 (10.01)	878 (10.7)
50+ yrs	21 (1.73)	23 (1.51)	91 (4.71)	86 (5.00)	66 (3.61)	287 (3.5)
Missing	130 (10.72)	254 (16.72)	83 (4.30)	59 (3.43)	11 (0.60)	537 (6.54)
Symptoms						
Fever	1129 (93.07)	1350 (88.87)	1864 (96.53)	1652 (96.16)	1803 (98.63)	7798 (95.00)
Cough	824 (67.93)	1099 (72.35)	1554 (80.47)	1393 (81.08)	1462 (79.98)	6332 (77.13)
Rhinitis	734 (60.51)	959 (63.13)	741 (38.37)	522 (30.38)	583 (31.89)	3539 (43.11)
Myalgia	125 (10.30)	327 (21.53)	306 (15.85)	302 (17.58)	273 (14.93)	1333 (16.24)
Pharyngitis	109 (8.98))	151 (9.94)	409 (21.18)	304 (17.69)	322 (17.61)	1295 (15.77)
headache	105 (8.66)	182 (11.98)	262 (13.57)	318 (18.51)	213 (11.65)	1080 (13.16)
dyspnea	20 (1.65)	24 (1.58)	87 (4.50)	23 (1.34)	37 (2.02)	191 (2.33)
vomiting	123 (10.14)	34 (2.24)	91 (4.71)	148 (8.61)	118 (6.45)	514 (6.26)
diarrhea	93 (7.67)	30 (1.97)	44 (2.28)	39 (2.27)	37 (2.02)	243 (2.96)
Asthenia	0 (0.00)	5 (0.33)	2 (0.10)	43 (2.50)	43 (2.35)	93 (1.13)

Among symptoms reported in patients, fever was the most common with 95% followed by cough (77.1%), rhinitis (43.1%), myalgia (16.2%), pharyngitis (15.8%), headache (13.2%), vomiting (6.3%) and diarrhea (3%). Dyspnea (2.3%) and asthenia (1.1%), reflecting a severe disease, have been observed in some patients.

### Detection of HMPV from patients with ILI

Of the 8209 respiratory specimens collected and tested by real-time RT-PCR, 308 were positive for HMPV, representing an overall prevalence of 3.7% (Table 2). The age of patients infected with HMPV varied from 1 month to 46 years with mean and median ages of 5 years 8 months and 2 years 4 months respectively. The male to female ratio of HMPV infected patients was 1.1 (163/144). Detection rate of HMPV over the years differ significantly (*p*-value = 1.225^e-08^). The highest detection rate was observed in 2012 (5.2%), while we noted a remarkable decrease of HMPV infection in 2015 (1.4%). Among the 308 HMPV positive samples, 89 (28.9%) were detected as single infection. The most common co-infecting viruses were influenza viruses accounting for 63 cases, followed by adenovirus with 41 cases, and rhinovirus with 23 cases. In 92 positive cases (29.9%), HMPV is detected with two or three more other respiratory viruses.

**Table 2:** Detection rates of Human metapneumovirus infection in patients with influenza-like illness per year from 2012 to 2016 in Senegal and comparison of the distribution in different age groups

year	2012	2013	2014	2015	2016	Total	*P*-values
Total tested	*N = 1213*	*N = 1519*	*N = 1931*	*N = 1718*	*N = 1828*	*N = 8209*	
Positivity n(%)	63 (5.20)	51 (3.36)	96 (4.97)	24 (1.40)	74 (4.05)	308 (3.75)	**1.225** ^**e-08**^
Gender,n(%)							
Male	29 (46.03)	24 (47.06)	45 (46.87))	14 (58.33)	51 (68.9)	163 (52.92)	1
Female	33 (52.38)	27 (52,94)	51 (53.12)	10 (41.66)	23 (31.1)	144 (46.75)	
Missing	1 (1.59)	0 (0.00)	0 (0.00)	0 (0.00)	0 (0.00)	1 (0.33)	
Age group, n(%)							
0–5 yrs	46 (73.01)	35 (68.63)	63 (65.62)	19 (79.16)	67 (90.54)	221 (71.75)	
5–10 yrs	7 (11.11)	5 (9.80)	8 (8.33)	1 (4.16)	7 (9.46)	29 (9.41)	
10–15 yrs	2 (3.17)	1 (1.96)	4 (4.16)	0 (0.00)	0 (0.00)	8 (2.59)	
15–25 yrs	0 (0.00)	3 (5.88)	9 (9.37)	2 (8.33)	0 (0.00)	16 (5.19)	**2.36** ^**e-11**^
25–50 yrs	1 (1.59)	0 (0.00)	11 (11.46)	1 (4.16)	0 (0.00)	15 (4.87)	
50+ yrs	0 (0.00)	0 (0.00)	0 (0.00)	0 (0.00)	0 (0.00)	0 (0.00)	
Missing	7 (11.11)	7 (13.72)	1 (1.04)	1 (4.16)	0 (0.00)	19 (6.17)	

*P*-values in bold are considered statistically significant

HMPV infections were observed in all age groups with exception to the elderly patients (≥50 years) (Table 2). However, the detection rates in these different age groups varied significantly (X^2^ = 61.379; *p*-value = 2.36^e-11^) with the children under 5 years group accounting for 71.7% (221/308) of HMPV positive patients. In the remaining groups, we observed a detection rate of 9.4% (29/308) in the 5-10 years age group, 2.6% in 10-15 years, 5.2% in adolescents (15-25 years) and 4.9% in adults (25-50 years).

### Clinical presentation of patients who are HMPV positive

Combining clinical symptoms and HMPV infection, the majority among the 308 HMPV positive patients experienced a fever (98.4%) which was the primary inclusion criterion (Table 3). Cough (89.6%; OR = 2.45; 95%CI 1.657–3.769; *p*-value = 8.094^e-07^), myalgia (10.4%; OR = 0.563; 95%CI 0.358–0.852; p-value = 0.00447) and headache (6.5%; OR = 0.444; 95%CI 0.253–0.730; p-value = 0.0005) were also significantly associated with HMPV infection.

**Table 3:** Human metapneumovirus infection and clinical signs in patients with Influenza like illness in Senegal from 2012 to 2016

Years	2012	2013	2014	2015	2016	Total	*P*-values
Total	*N = 1213*	*N = 1519*	*N = 1931*	*N = 1718*	*N = 1828*	*N = 8209*	
Positivity (per year)	63 (5.20)	51 (3.36)	96 (4.97)	24 (1.40)	74 (4.05)	308 (3.75)	**1.225** ^**e-08 b**^
clinical signs,n(%)							
Fever	58 (92.06)	51 (100)	96 (100)	24 (100)	74 (100)	303 (98.37)	**0.03828** ^**a**^
Cough	53 (84.13)	44 (86.27)	86 (89.58)	24 (100)	69 (93.24)	276 (89.61)	**8.094** ^**e-07 a**^
Rhinitis	38 (60.32)	40 (78.43)	31 (32.29)	5 (20.83)	10 (13.51)	124 (40.25)	0.6542^a^
Myalgia	5 (7.93)	7 (13.72)	12 (12.5)	3 (12.5)	5 (6.75)	32 (10.38)	**0.00447** ^**a**^
Pharyngitis	6 (9.52)	5 (9.80)	15 (15.62)	2 (8.33)	7 (9.46)	35 (11.36)	0.09454^a^
headache	3 (4.76)	4 (7.84)	7 (7.29)	2 (8.33)	4 (5.4)	20 (6.49)	**0.0005** ^**a**^
dyspnea	1 (1.59)	1 (1.96)	3 (3.12)	0 (0.00)	0 (0.00)	5 (1.62)	1^a^
vomiting	7 (11.11)	0 (0.00)	6 (6.25)	6 (25)	9 (12.16)	28 (9.09)	0.06528^a^
diarrhea	6 (9.52)	0 (0.00)	6 (6.25)	0 (0.00)	0 (0.00)	12 (3.89)	0.1429^a^
Asthenia	0 (0.00)	0 (0.00)	0 (0.00)	0 (0.00)	2 (2.7)	2 (0.64)	0.7308^a^

*P*-values in bold are considered statistically significant

^a^Fisher’s exact test, ^b^ Chi-square test

### Seasonal distribution of HMPV

**Fig. 1:**
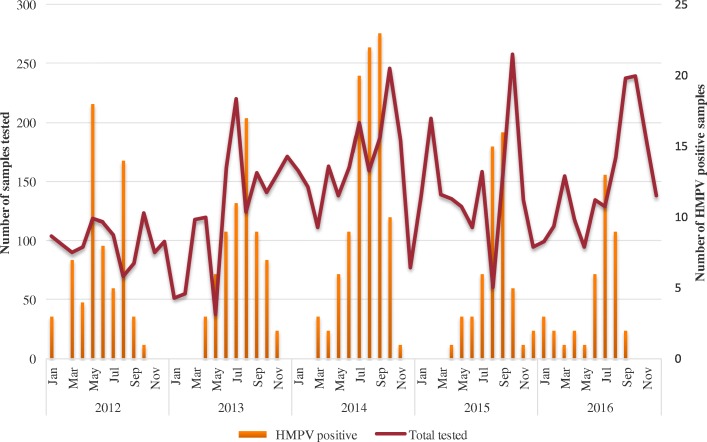
Distribution of adenovirus-positive cases among patients with influenza-like illness, by month and year. The curve represents the total number of influenza-like Illness cases tested for HMPV. Bars represent the number of HMPV-positive cases for each month

Next, we analyzed the temporal distribution pattern on a monthly basis for HMPV infection (Fig. 1). HMPV occurrence showed a clear seasonal pattern. Indeed, for all years, we observed a systematic increase of HMPV infections in the population from May through September, with a peak mapped in July–August period excepted in 2012 where we observed an earliest peak in May. These periods of higher activity of HMPV coincide with the rainy seasons in Senegal.

### Phylogenetic analysis and typing of HMPV

**Fig. 2:**
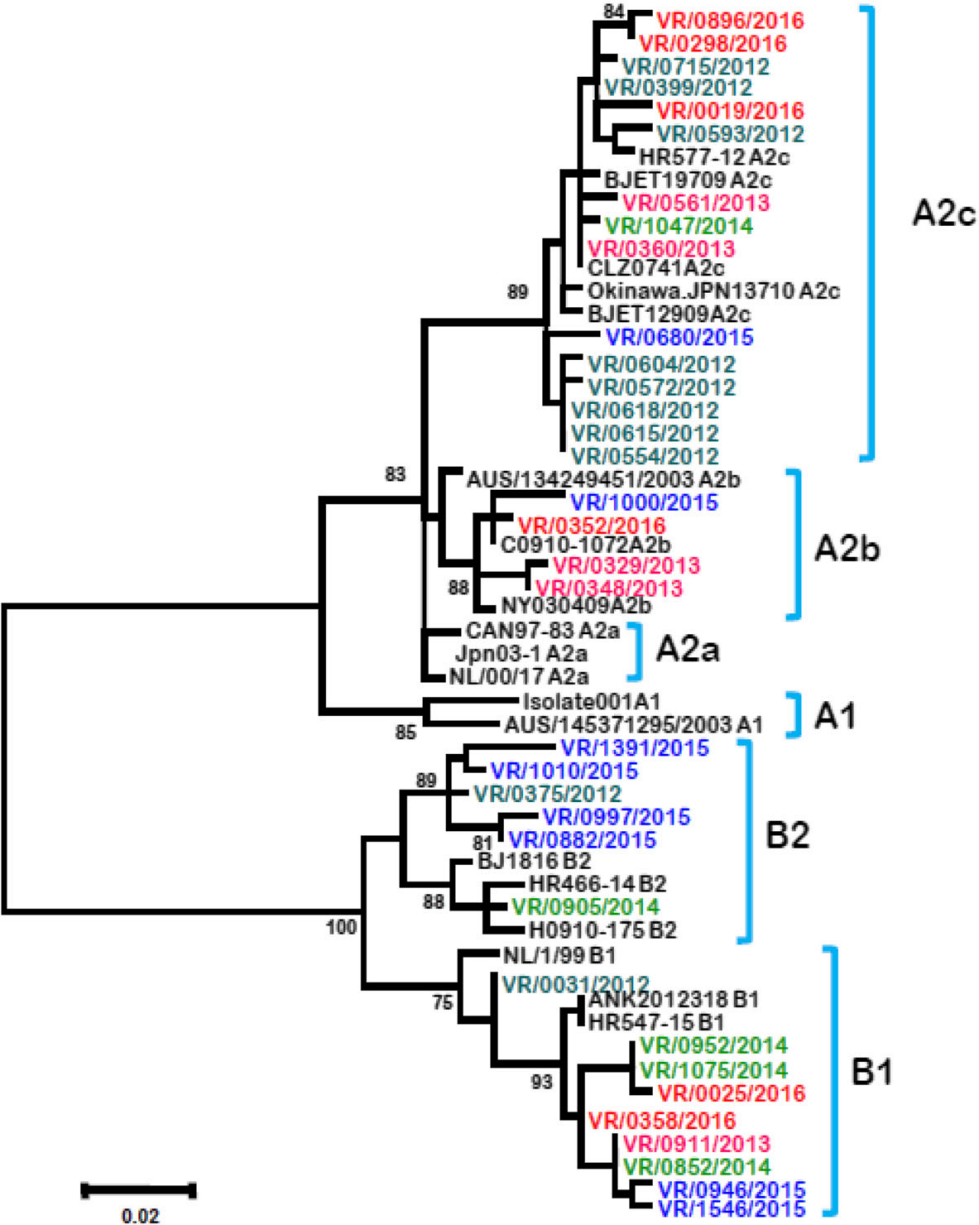
phylogenetic analyses of human metapneumovirus (HMPV) strains detected in patients with ILI between 2012 and 2016 in Senegal. The phylogenetic tree based on nucleotide sequences of partial Fusion protein gene was generated using the neighbor-joining method, as implemented in MEGA 6 software. 1000 bootstrap replicates were performed to determine the consensus tree presented in this figure, support for nodes present in greater than 70% of the trees are annotated. Reference strains used in the analysis are represented in black color and Senegalese strains are represented in different colors depending on the year of detection

We successfully obtained the partial F gene sequence from 35 HMPV positive samples: 9 from samples in 2012, 6 from 2013, 4 from 2014, 9 from 2015 and 5 from 2016. Phylogenetic analyses revealed that these 35 HMPV specimens were distributed into the two main genetic lineages: lineage A with 19 strains and the lineage B with 16 strains (Fig. 2). Of the 19 A lineage specimens, 15 were grouped with the A2c subtype and 4 with the A2b subtype, while the B lineage specimens included 9 clustering with the B1 subtype and 7 with the B2 subtype reference strains. No strain belonging to subgroups A1 and A2a was sequenced in this study.

**Fig. 3:**
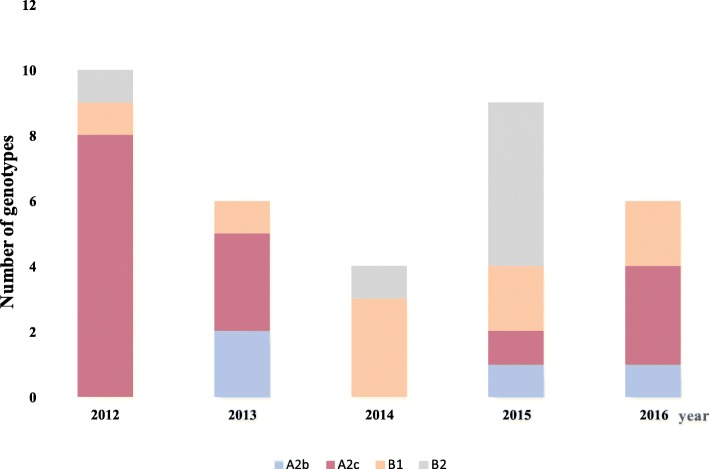
Distribution of circulating genotypes per year in patients with Influenza like illness in Senegal between 2012 and 2016

We also noted a co-circulation of both genetic lineages during the whole study period except in 2014 where subgroup B strains exclusively circulated with the majority belonging to B1 (75%) genotype. Indeed, in 2012 genotypes A2c, B1 and B2 co-circulated with the A2c genotype accounting for 80% (8/10), while in 2013 and 2016 genotypes A2b, A2c and B1 co-circulated with the majority of the strains belonging to A2c genotype. In 2015, the same subgroups (A2b, A2c and B1) co-circulated with the predominating B2 genotype (Fig. 3).

## Discussion

The present study reported the epidemiological profile of HMPV based on an outpatient population of adults and children presented with symptoms of ILI in Senegal during a five-year period of sentinel surveillance.

From 8209 nasopharyngeal swabs collected between 2012 and 2016, the overall prevalence of HMPV was 3.75%, similar to rates reported in previous studies conducted in other countries including Egypt with 4% [13], in Greece 3.7% [14], in the United States with 3.6% [15] and in India 3.6% [16]. Lower detection rates have even been reported in studies conducted in other geographical areas, such as Cameroon [17], Cambodia [18], and Malaysia [19] with respectively 2.3, 1.7 and 1.1% of prevalence. Nevertheless, other studies have reported much higher detection rates; this is the case in Taiwan where a prevalence of 23% [20] was reported in a study conducted between 2005 and 2010, or in Jordan with 12.7% [21]. The discrepancies in HMPV detection rates among patients with ILI in different areas highlighted geographical differences in virus burden. However, these geographical differences reported with regard to the detection rates should have been accentuated by several other factors such as different technical approaches, the sampling period, the study duration, or the targeted population (global population, pediatric ...).

Regarding the detection rate of HMPV per age, the virus has been detected in all age groups with exception to elderly patients. The non detection of HMPV in this age group would be probably due to the low number of patients (3.5% of overall patients) from this group as a previous study exclusively focused on the elderly revealed a HMPV detection rate of 3.3% [9]. In our study, the majority (71.8%; 221/308) of HMPV-positive patients were children between 0 to 5 years old, with more than the half of them under 1 year age (55.7%, 123/221). Consistent with our findings, several studies have reported this sensitivity of children under 5, especially in the first year life, to HMPV infection [22–24]. This highest prevalence in children is believed to be due to a naive immune system for most of HMPV genotypes in early childhood. The bias in the composition of our study population (nearly 50% of children under 5) is another factor that would have accentuated the trend noted. No influence of the patients’ gender on HMPV infection was observed in our study, which is consistent with the results of a study in China [25]. However, sex discrimination had been previously reported [26, 27]. Our findings regarding Influenza, HAdV and HRV as the most frequently co-detected viruses with HMPV were similar to those of Kong et al. [25]. Fever, cough, myalgia, and headache were the predominant clinical features associated with HMPV detection, which is in line with results from a study conducted in China [28].

The circulation pattern of HMPV during these 5 years of surveillance showed a clear seasonality of HMPV infection in Senegal with a highest activity of the virus between May and November. This period coincide with the rainy season in Senegal with a relatively high temperature and humidity. This is consistent with studies conducted in Cameroon [29], Cambodia [18], India [30] and Thailand [23] where peaks of HMPV infections had been recorded during rainy season’s periods. The circulation pattern of the HMPV between 2012 and 2015 is also similar to that of RSV in the same period [31]. Both viruses are enveloped and the relatively high humidity during the rainy season seems to be an advantage for viruses’ persistence in the air.

We were able to obtain the partial F gene sequence from 35 HMPV positive samples. Indeed, many samples showed no amplification or poor-quality sequences. The low sensitivity of conventional PCR compared with real time PCR on samples with low viral load, and certainly non-specific amplifications could be the cause of these failures. Phylogenetic analysis demonstrated co-circulation of mainly three HMPV subgroups A2, B1 and B2. A similar genotypic composition has been observed in several other studies [13, 32, 33]. And as in other studies conducted in Saudi Arabia [26], in Croatia [34], in Panama [24] or in Kenya [32], we have no strain belonging to genotype A1, supporting the idea that old lineages have been replaced by emerging genetic strains. Indeed, the A2c subgroup was present in Senegal throughout the study period, except in 2014 where it was not detected probably due to a non-exhaustive sequencing. The A2c genotype was the dominant circulating genotype in 2012–2013 periods, and then B1 in year 2014 where all the sequences obtained belonged exclusively to genotype B. However, in 2015 we observed a co-circulation of 4 genotypes with a predominance of subgroup B2 while A2c subgroup dominated in 2016. These results, despite the small number of sequenced strains, confirm previous observations on the changes and predominance of circulating HMPV genotypes [35].

We observed some limitations in our study. First, only a small number of HMPV were typed. So the sequencing results do not reflect the full spectrum of HMPV genotypes that may circulate in ILI patients in Senegal, and even for selected samples it may have a bias toward samples with a high viral load. Also it would be interesting to analyze G gene region coding for attachment proteins for more acuity on the genetic variability of circulating strains. Secondly, in our study only outdoor patients were recruited. Thus further studies including both outpatients and inpatients (hospitalized) would be required to firmly establish the burden associated with HMPV in Senegal and correlations between disease severity and genotypes.

## Conclusion

In summary, the present study characterized the recent prevalence, seasonality and genetic diversity of HMPV in a large outpatient population presented with ILI in Senegal between 2012 and 2016. Globally our results showed a clear seasonal pattern of HMPV in the second half of each year, between June and October with a possibly extending into November. Our findings also identified children less than 5 years old as being more susceptible to HMPV infection. Molecular studies identified A2, B1 and B2 as the major genotypes circulating. However, additional sequencing work targeting both G and F genes should be carried out in order to enhance understanding of the local and global molecular epidemiology of HMPV strains from Senegal. The next step will be the the HMPV burden assessment in Senegal, especially in pediatric hospitalized cases. Data on disease outcome, atypical clinical signs, viral load, shedding, duration of symptoms, duration of hospitalization and treatment will be collected and included in analyses.

## Abbreviations

ARI: acute respiratory infection
CDC: Center of Disease Control
cDNA: Complementary deoxyribonucleic acid
DNA: deoxyribonucleic acid
F protein/gene: fusion
HAdV: Human adenovirus
HMPV: human metapneumovirus
HRV: Human rhinovirus
ILI: Influenza-like illness
IPD: Institut Pasteur Dakar
NIC: National Influenza Center
PCR: polymerase chain reaction
RNA: ribonucleic acid
RSV: Respiratory syncytial virus
UV: ultra-violet
WHO: World Health Organization
